# Persistence of Human Bocavirus 1 in Tonsillar Germinal Centers and Antibody-Dependent Enhancement of Infection

**DOI:** 10.1128/mBio.03132-20

**Published:** 2021-02-02

**Authors:** Man Xu, Maria Fernanda Perdomo, Salla Mattola, Lari Pyöriä, Mari Toppinen, Jianming Qiu, Maija Vihinen-Ranta, Klaus Hedman, Johanna Nokso-Koivisto, Leena-Maija Aaltonen, Maria Söderlund-Venermo

**Affiliations:** a Department of Virology, University of Helsinki, Helsinki, Finland; b Department of Biological and Environmental Science, University of Jyväskylä, Jyväskylä, Finland; c Nanoscience Center, University of Jyväskylä, Jyväskylä, Finland; d Department of Microbiology, Molecular Genetics and Immunology, University of Kansas Medical Center, Kansas City, Kansas, USA; e Helsinki University Hospital, Helsinki, Finland; f Department of Otorhinolaryngology-Head and Neck Surgery, Helsinki University Hospital, Helsinki, Finland; g University of Helsinki, Helsinki, Finland; Yale University School of Medicine; McMaster University

**Keywords:** parvovirus, germinal center, tonsils, virus persistence, ADE, *in situ* hybridization

## Abstract

Human bocavirus 1 (HBoV1), a nonenveloped single-stranded DNA parvovirus, causes mild to life-threatening respiratory tract infections, acute otitis media, and encephalitis in young children. HBoV1 often persists in nasopharyngeal secretions for months, hampering diagnosis. It has also been shown to persist in pediatric palatine and adenoid tonsils, which suggests that lymphoid organs are reservoirs for virus spread; however, the tissue site and host cells remain unknown. Our aim was to determine, in healthy nonviremic children with preexisting HBoV1 immunity, the adenotonsillar persistence site(s), host cell types, and virus activity. We discovered that HBoV1 DNA persists in lymphoid germinal centers (GCs), but not in the corresponding tonsillar epithelium, and that the cell types harboring the virus are mainly naive, activated, and memory B cells and monocytes. Both viral DNA strands and both sides of the genome were detected, as well as infrequent mRNA. Moreover, we showed, in B-cell and monocyte cultures and *ex vivo* tonsillar B cells, that the cellular uptake of HBoV1 occurs via the Fc receptor (FcγRII) through antibody-dependent enhancement (ADE). This resulted in viral mRNA transcription, known to occur exclusively from double-stranded DNA in the nucleus, however, with no detectable productive replication. Confocal imaging with fluorescent virus-like particles moreover disclosed endocytosis. To which extent the active HBoV1 GC persistence has a role in chronic inflammation or B-cell maturation disturbances, and whether the virus can be reactivated, will be interesting topics for forthcoming studies.

## Abstract (precis)

Human bocavirus 1 (HBoV1), a common pediatric respiratory pathogen, can persist in airway secretions for months hampering diagnosis. It also persists in tonsils, providing potential reservoirs for airway shedding, with the exact location, host cell types, and virus activity unknown.

## INTRODUCTION

Human bocavirus 1 (HBoV1), a small nonenveloped linear single-stranded DNA virus of the *Parvoviridae* family, was discovered in 2005 in nasopharyngeal aspirates of children with respiratory tract infections (RTI) (1). Accumulating evidence has shown that HBoV1 causes both upper and lower respiratory tract infections of diverse severity and affects most children before age 7 (2, 3). After primary infection, HBoV1 can, despite a vigorous antibody response, persist in the respiratory tract for at least up to 12 months (4–7), explaining the frequent codetection of HBoV1 with other viruses, hampering diagnosis. Several studies have documented intermittent excretion of HBoV1 DNA (7–10), suggesting reinfection or reactivation by an unknown source and mechanism.

HBoV1 DNA has frequently been detected in both palatine and adenoid (nasopharyngeal) tonsils of children with chronic tonsillitis and hypertrophy but without symptoms of RTI (11–18). Tonsils could thus be a reservoir for virus spread. Indeed, tonsillectomy seems to reduce the excretion of HBoV1 (19, 20). Notwithstanding the evidence of HBoV1 persistence in tonsils and adenoids, the specific tissue site, cell type(s) harboring the virus, and virus activity, are unknown. Lu et al. found HBoV1 PCR positivity in Ficoll-Paque-separated tonsillar mononuclear cells in 32% of the children, without further characterization of cell types or virus activity (12).

HBoV1 has been shown to productively infect differentiated air-liquid interface cell cultures of human airway epithelium (HAE) (21, 22). In young children, HBoV1 primary infection is therefore thought to target the airway epithelial cells. Yet, how the virus infects lymphoid tissues remains unknown. In Aleutian mink parvovirus infection of permissive macrophages and parvovirus B19 (B19V) infection of presumably nonpermissive monocytes, B cells, and endothelial cells, the virus uptake has been shown to occur by antibody-dependent enhancement (ADE) (23–26).

Our aim was to characterize HBoV1 infection and persistence in adenotonsillar tissues. We observed the persistent HBoV1 DNA in adenoids to localize exclusively in germinal centers (GCs) and the cell types harboring the virus to be mainly B cells and monocytes. Furthermore, in human B-cell and monocyte cultures, as well as *ex vivo*-cultured tonsillar B cells, we demonstrated HBoV1 virions to enter cells by antibody-dependent enhancement via the cellular Fc gamma receptor (FcγRII), leading to mRNA transcription, indicating that synthesis of double-stranded DNA (dsDNA) had occurred and the virus had been transported to the nucleus. We further showed that fluorescent virus-like particles (VLPs) localize in B-cell endosomes. Nevertheless, no productive infection was observed.

## RESULTS

### HBoV1 DNA persistence in tonsils and cell fractions.

**FIG 1:**
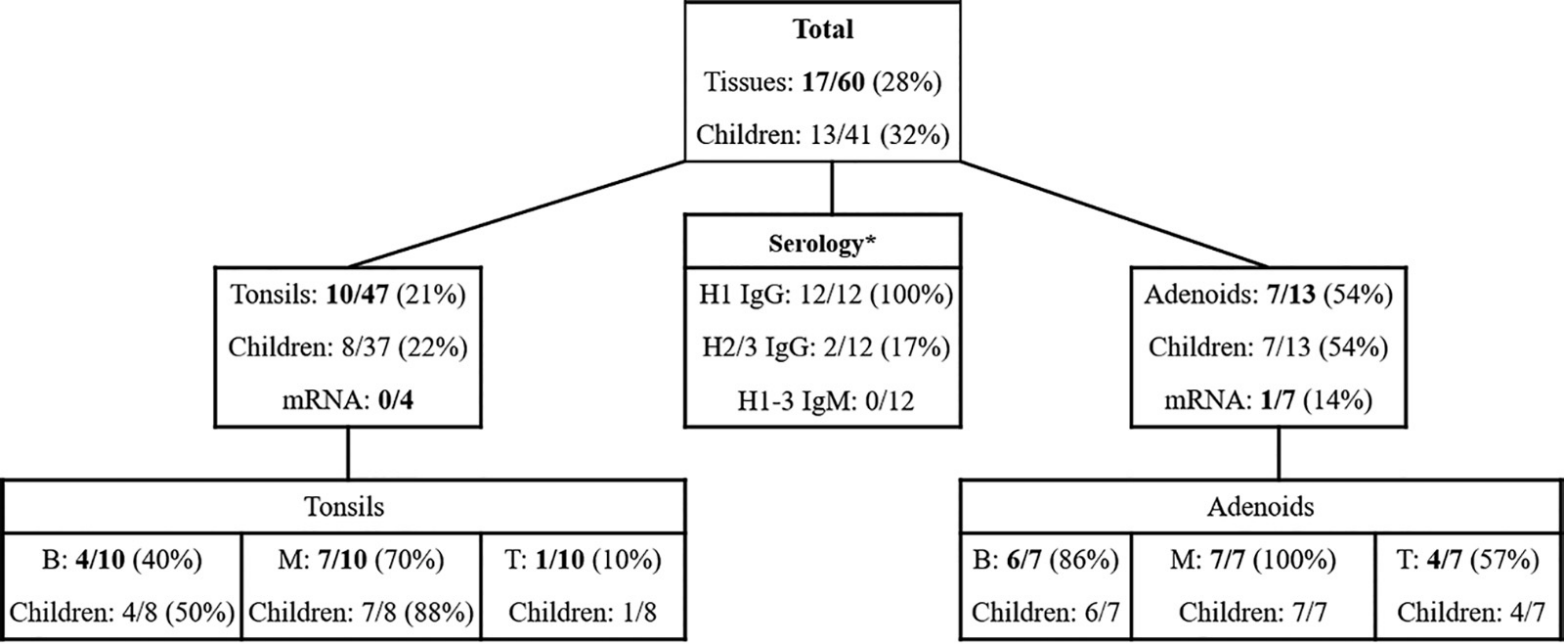
HBoV1 DNA and mRNA in palatine tonsils and adenoids of children below 10 years of age, DNA in their magnetic bead-separated cell fractions, as well as serology of the HBoV1 DNA-positive children. The asterisk after Serology indicates that 12/13 HBoV1 DNA-positive children had available serum. All separated cell fractions were mRNA negative. IgG/M, immunoglobulin G/M; H1-3, human bocavirus 1 to 3; B, B-cell fraction; M, monocyte fraction; T, T-cell fraction.

We searched for and quantified HBoV1 DNA with quantitative PCR (qPCR), targeting the left side (*NS1* gene) of the genome, in total DNA preparations (from tissue preparation 1 [Prep 1]) of 98 tonsillar tissues from 79 individuals. Among the >10-year-old patients with chronic tonsillitis or tonsillar hypertrophy, only 1 of 38 (2.6%) tonsils was positive for HBoV1 DNA (a tonsil of a 34-year-old male). Among the children ≤10 years of age, 13 of 41 (32%) were positive for HBoV1 DNA in tonsil, adenoid, or both (Fig. 1). HBoV1 DNA was detected in 54% (7/13) of adenoids, 21% (10/47) of individual tonsils, and 22% (8/37) of tonsil pairs per child. Overall, the viral DNA loads were low (median, 5.9 × 10^1^ copies/10^6^ cells; range, 2.6 × 10^0^ to 2.9 × 10^6^ copies/10^6^ cells). Among nine patients with both tonsillar and adenoidal tissues available, HBoV1 DNA was detected in the adenoids alone in two patients, in the tonsils alone in no patients, and in both adenoids and tonsils (of both left and right sides) in two patients, with 24- and 5,900-fold-higher viral loads in adenoids than in tonsils. Another qPCR, amplifying the right side (*VP* gene) of the HBoV1 genome, yielded similar viral loads (see Fig. S1 in the supplemental material).

10.1128/mBio.03132-20.2 FIG S1 Correlation in HBoV1 DNA copies quantified with NS1 qPCR and VP qPCR from HBoV1 DNA-positive tissues of 2019. Download FIG S1, TIF file, 1.0 MB. Copyright © 2021 Xu et al. 2021 Xu et al. This content is distributed under the terms of the Creative Commons Attribution 4.0 International license.

B-cell, T-cell, and monocyte fractions were enriched with magnetic beads from all 17 HBoV1 DNA-positive tonsillar tissues (10 tonsils and 7 adenoids) collected from 13 children. The tonsillar tissues comprised 65.2% B cells, 2.1% monocytes, and 22.9% T cells; of the latter, 75% were CD4^+^ Th cells (Fig. S2A). Viral DNA was detected in 14/17 (82%) monocyte/macrophage fractions, in 10/17 (59%) B-cell fractions, and 5/17 (29%) T-cell fractions. Of note, all 7 HBoV1 DNA-positive adenoids but only 7 of 10 (70%) tonsils had virus detectable in at least one corresponding cell fraction (Fig. 1).

10.1128/mBio.03132-20.3 FIG S2 Tonsillar cell fractions and cellular Fc receptor (FcγR) staining. (A) Cell preps from 7 tonsils were stained for B cells, T cells, and monocytes with distinct cellular markers (B: CD20, monocytes: CD14, T: CD3, Th, helper T: CD4). (B) FcγR staining of *ex vivo*-isolated tonsillar B cells, B and T cells, and monocytes with anti-FcγRII (CD32) or anti-FcγRI (CD64) antibodies. Cells were analyzed on a BD Accuri C6 flow cytometer. Download FIG S2, TIF file, 1.1 MB. Copyright © 2021 Xu et al. 2021 Xu et al. This content is distributed under the terms of the Creative Commons Attribution 4.0 International license.

A 3-year-old asymptomatic child had a very high viral load (2.9 × 10^6^ copies/10^6^ cells) in his adenoid tissue, as well as in the isolated monocyte (9.5 × 10^6^ copies/10^6^ cells), B-cell (2 × 10^4^ copies/10^6^ cells), and T-cell (2.8 × 10^4^ copies/10^6^ cells) fractions. In contrast, his left and right tonsils and their subpopulations had lower HBoV1 DNA loads (≤4.9 × 10^2^ copies/10^6^ cells).

### Viral transcription in tonsillar tissues.

The one high-load adenoid (of Prep 1) described above was repeatedly low positive for HBoV1 *NS1* mRNA (Fig. 1) (quantification cycle [Cq] = 36 to 37 with reverse transcription-PCR [RT-PCR]), whereas *NP1* and *VP* mRNAs were below detection level. In the *NS1* RT-PCR, the DNase I-treated RNA prep (no-reverse transcriptase [RT] control) was negative, ruling out interfering DNA amplification. All the bead-enriched cell fractions from this adenoid were, however, negative for all the viral mRNAs (*NP*, *VP*, and *NS1*). Neither were the viral mRNAs detectable in the remaining 10/11 tissues with lower HBoV1 DNA loads.

### Prior HBoV1 immunity in the children.

Twelve of the 13 HBoV1 DNA-positive children had HBoV1-specific IgG antibodies, yet without IgM, indicating preexisting immunity (Fig. 1). From the remaining child, a serum sample was not available. The overall HBoV1 seroprevalence of the children ≤10 years of age was 74.4%. The 3-year-old child described above was nonviremic and exhibited long-term HBoV1 immunity (positive for HBoV1 IgG, negative for HBoV1 IgM). Furthermore, he was seronegative for HBoV2 and HBoV3. Altogether, only 2 children among the 12 HBoV1 DNA-positive children with available serum were HBoV2 or 3 IgG positive (Fig. 1).

### HBoV1 DNA persistence in tonsillar germinal centers.

**FIG 2:**
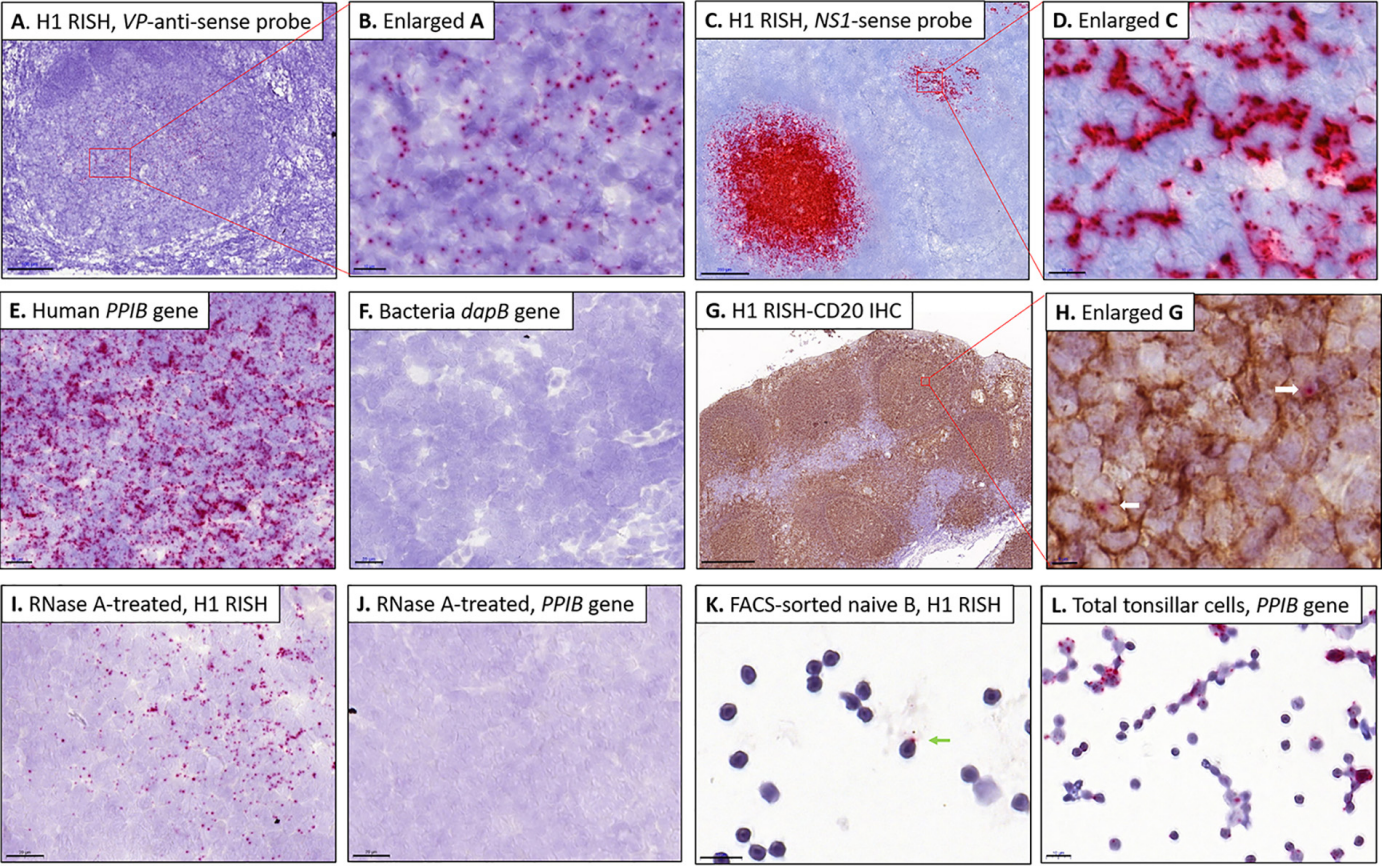
HBoV1 staining of germinal centers (GCs) of adenoid sections and FACS-sorted naive B cells by RNAscope ISH (RISH). Red dots represent HBoV1 DNA/mRNA signals. The sections were counterstained with hematoxylin (blue). (A and B) The high-load adenoid stained with *VP* antisense probe showing HBoV1 positive-strand DNA/mRNA staining in the GCs (A) and a higher-magnification view (B). (C and D) The high-load adenoid stained with *NS1* sense probe showing one exceptionally dark stained GC with HBoV1 negative-strand DNA (C). A more typical GC is shown magnified (D). Note that this sense probe gives, for unknown reasons, wider and more saturated red dots than the *VP* probe. (E and F) Human reference gene *PPIB* and bacterial gene *dapB* as positive and negative controls, respectively. (G and H) IHC staining of the B-cell marker CD20 in one adenoid following HBoV1 RISH (G) and a higher-magnification view of panel G (H). Arrows indicate the localization of virus. (I and J) HBoV1 *VP* antisense-probed tissue (I) and *PPIB*-probed tissue (J), treated with RNase A. (K and L) One B cell (arrow) showing HBoV1 staining in naive B cells sorted from one high viral load adenoid (K), and a few B cells showing *PPIB* staining in a total tonsil cell suspension (L). Bars, 500 μm (G), 200 μm (C), 100 μm (A), 20 μm (E, F, I, and J), 10 μm (B, D, K, and L), and 5 μm (H). H1, human bocavirus 1 (HBoV1); GC, germinal center; *PPIB*, human housekeeping gene, positive-control probe; *dapB*, bacterial gene, negative-control probe; RISH, RNAscope *in situ* hybridization; IHC, immunohistochemistry.

To further pinpoint the persistence site of HBoV1 in tonsillar tissue, RNAscope *in situ* hybridization (RISH) targeting HBoV1-*VP* positive-strand DNA and mRNA, was applied to fresh-frozen OCT-embedded tissue sections (Prep 2). All four adenoids with medium to high viral loads (3.5 × 10^3^ to 2.9 × 10^6^ copies/10^6^ cells) showed by RISH the presence of HBoV1 DNA, indicated as red dots in Fig. 2A and B. The viral DNA was not dispersed throughout the adenoid tissues; instead the signals were exclusively confined to the round germinal centers (GCs). The absolute numbers of infected cells per GC ranged from 10 to 500 with one to three dots visible per cell. In contrast, no virus was observable by *VP* RISH in the seven tonsils or three adenoids with low-load HBoV1 DNA. To ensure that the cells harbored both left and right sides of the viral genome, as well as both of the DNA strands, three of the four HBoV1 *VP* RISH-positive adenoids were further restained with *NS* sense probes targeting the negative DNA strand on sequential tissue sections, with positive results. This *NS* RISH also confirmed the exclusive GC localization of HBoV1, with even stronger signals (Fig. 2C and D). *NS* RISH furthermore stained the GCs of one of the two low-viral-load tonsils of the above-mentioned 3-year-old child. The positive and negative technical control probes worked appropriately (Fig. 2E and F). Immunohistochemistry (IHC) with the B-cell marker CD20 (diaminobenzidine [DAB] brown) following HBoV1 *VP* RISH substantiated that the virus is located in the GCs (Fig. 2G and H).

In addition, HBoV1 *VP* RISH of RNase A-treated sections disclosed that the signal mainly originated from viral DNA rather than mRNA (Fig. 2I), indicating the presence of positive-strand viral DNA of this generally negative-strand single-stranded DNA (ssDNA) virus. Further, most of the signal remained after RNase treatment also with the *NS* sense probe, as expected (data not shown). The housekeeping gene (*PPIB*) RISH mRNA control showed no staining in sections similarly treated with RNase A (Fig. 2J).

### Tonsillar B-cell subpopulation infected with HBoV1.

**FIG 3:**
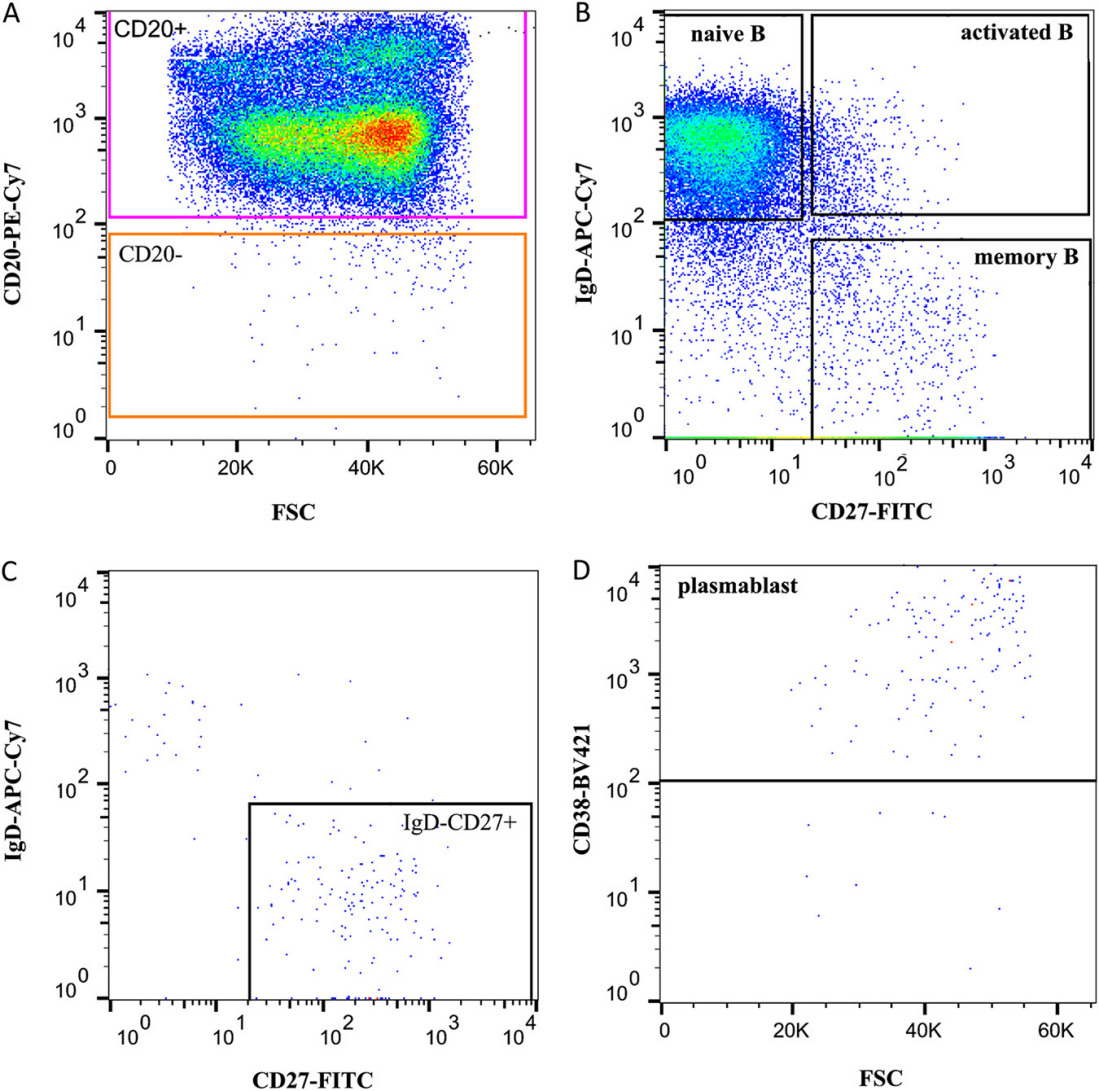
FACS sorting of B-cell subpopulations from one representative adenoid. (A) Total cells and singlets were selected, dead cells were excluded by 7AAD staining, and total B cells were identified from CD19^+^ stained cells and gated based on the expression of CD20. (B) CD20^+^ B cells from panel A were further plotted for the expression of IgD and CD27, which separate three subpopulations: naive B (IgD^+^ CD27^−^), activated B (IgD^+^ CD27^+^), and memory B cells (IgD^−^ CD27^+^). Additionally, IgD^−^ CD27^+^ B cells (C) were gated from CD20^−^ subpopulation (A) and then further gated for CD38^+^ plasmablasts (D). FACS, fluorescence-activated cell sorting; FSC, forward scatter.

**FIG 4:**
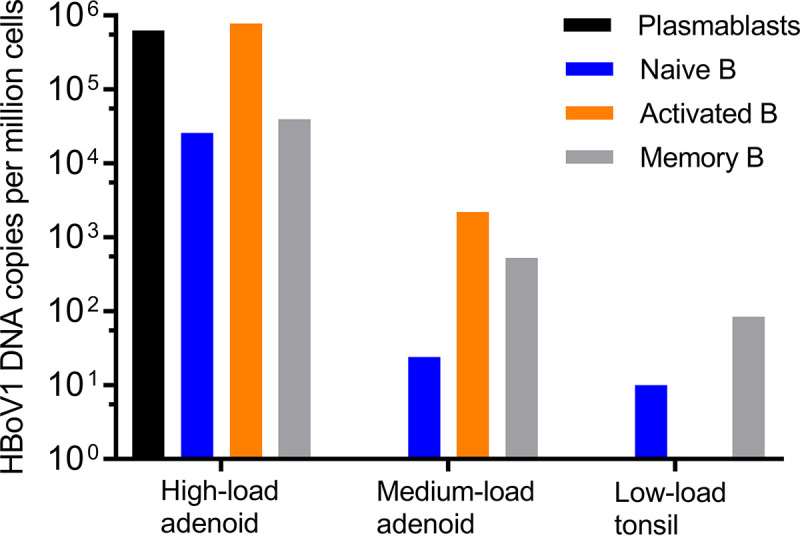
HBoV1 DNA presence in B-cell subsets (plasmablasts and naive, activated, and memory B cells) sorted from two adenoids and one tonsil. Viral loads are presented as copies per one million cells.

From the HBoV1 DNA-positive tissues, four adenoids and two tonsils (Prep 3) were sorted by fluorescence-activated cell sorting (FACS) for B-cell subpopulations. The gating strategy is illustrated in Fig. 3. Viral DNA was found by qPCR mainly in naive and memory B cells of three adenoids and one tonsil and also in activated B cells of two adenoids (median, 4.3 × 10^3^ copies/10^6^ cells; range, 1 × 10^1^ to 7.8 × 10^5^ copies/10^6^ cells). One low-load tonsil showed no HBoV1 DNA in any B-cell subpopulation, whereas one high-load adenoid showed HBoV1 DNA in all four subpopulations (median, 3.3 × 10^5^ copies/10^6^ cells; range, 2.6 × 10^4^ to 7.8 × 10^5^ copies/10^6^ cells). Viral DNA results in B cells of two representative adenoids and one tonsillar tissue sample are shown in Fig. 4. However, no significant differences in viral loads were noted between the B-cell subpopulations. HBoV1 *VP* RISH of the sorted B-cell subpopulations showed HBoV1 staining only in the high-load adenoidal naive B cells (Fig. 2K). *PPIB* positive-control RISH in total tonsil cells is shown in Fig. 2L.

### Antibody-dependent enhancement of HBoV1 infection.

**FIG 5:**
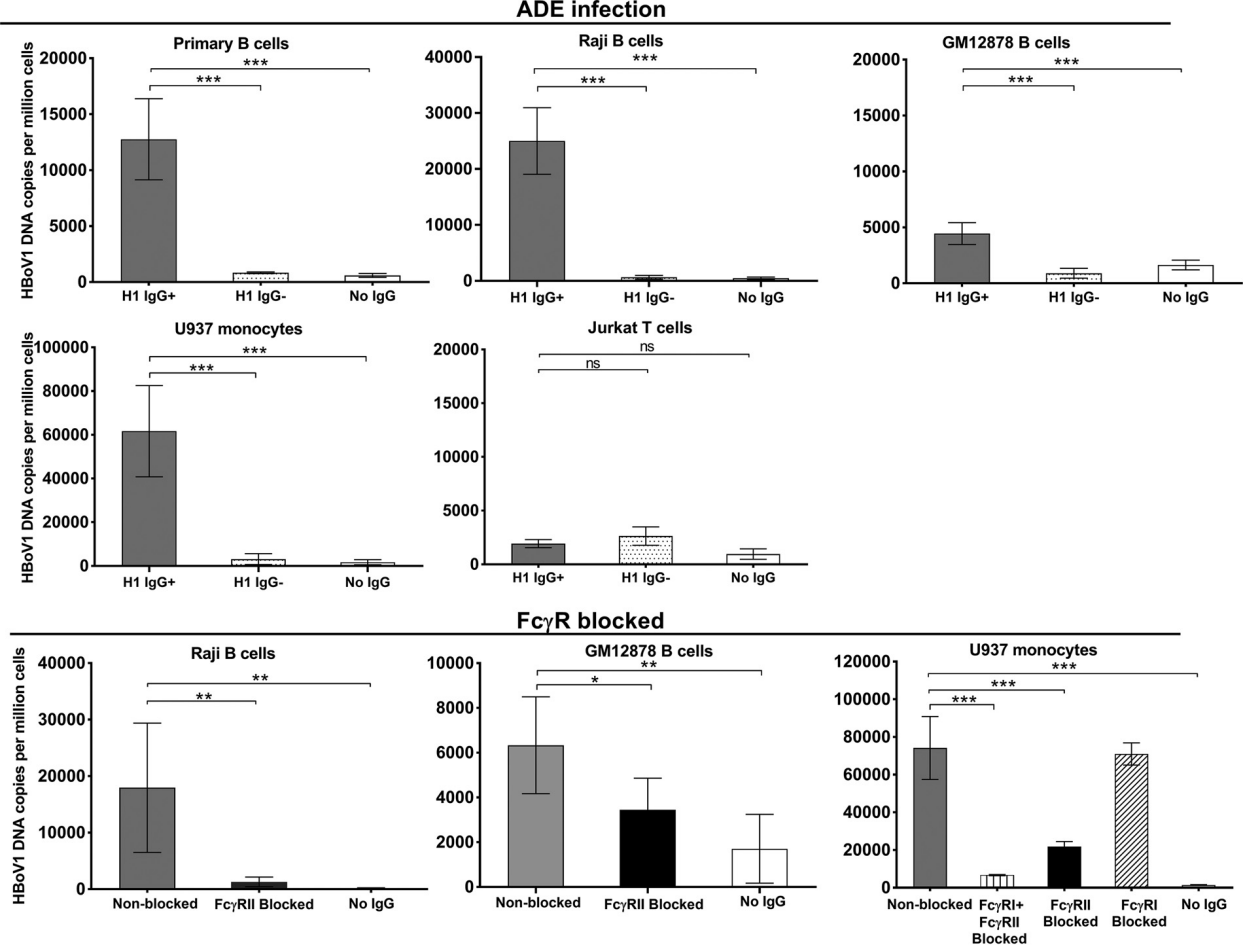
Antibody-dependent enhancement (ADE) of HBoV1 infection in *ex vivo*-isolated tonsillar B cells, monocyte (U937), T-cell (Jurkat). and two B-cell (Raji and GM12878-B) cultures, as well as blocking of Fc receptors of monocyte and two B-cell lines. Cells were infected at an MOI of 100 genome copies per cell for 2 days in the presence of 10 μg/ml total purified IgG with or without HBoV1 reactivity: HBoV1-positive (H1 IgG^+^) or -negative (H1 IgG^−^) IgG. For blocking of Fc receptors, cells were incubated with either anti-FcγRII (CD32) or anti-FcγRI (CD64) antibodies or both before infection. Statistical significance by Student’s *t* test is indicated as follows: ns, not significant; *, *P* < 0.05, **, *P* < 0.01, ***, *P* < 0.001. Please note the different scales of the *y* axes.

The possibility of antibody-dependent enhancement (ADE) of HBoV1 infection was determined with *ex vivo* tonsillar B cells, two human B-cell lines (GM04671-Raji and GM12878), and one monocyte line (U937), all expressing high levels of FcγRII on their cell surface, and T cells (Jurkat), expressing close to no FcγRII (Fig. S2B). The presence of HBoV1 IgG antibodies significantly enhanced the uptake of virus in all B cells and monocytes, evidenced by a 10- to 100-fold elevation of the viral loads (*P* < 0.001, Student’s *t* test) but had no effect on the T cells (Fig. 5).

Furthermore, in Raji B cells, HBoV1-VLP-Alexa Fluor 488 fluorescence was significantly enhanced during 8 h by the presence of HBoV1 IgG-containing serum (*P* < 0.01, Student’s *t* test) (Fig. S3A). No significant difference in viral uptake was seen between heated and nonheated serum, ruling against a role of complement in ADE (Fig. S3B). Moreover, ADE was verified by a significant block of viral uptake in the two B-cell lines and monocytes treated with mouse anti-human CD32 (FcγRII) or CD64 (FcγRI) antibodies (*P* < 0.05, Student’s *t* test, Fig. 5). As opposed to these cells, HBoV1-specific antibodies showed strong neutralization of the virus in permissive HAE cells (Fig. S4).

10.1128/mBio.03132-20.4 FIG S3 Uptake of fluorescent VLPs (A) or HBoV1 virions (B) into Raji cells in the presence of serum pools. (A) Raji cells were incubated with HBoV1 VLPs-Alexa Fluor 488 for 1 to 24 h in the presence of HBoV1 IgG-positive (H1 IgG^+^) or -negative (H1 IgG^−^) serum pools (heat-inactivated serum; 1:100 diluted), the mean fluorescence intensity was measured by flow cytometer, and triplicates are shown. (B) Raji cells were infected for 2 days in the presence of either heat-inactivated or non-heated H1 IgG^+^ or H1 IgG^−^ serum pools (1:100 diluted). Statistical significance by Student’s *t* test: ns, not significant; **, P < 0.01; ***, P < 0.001. Download FIG S3, TIF file, 2.1 MB. Copyright © 2021 Xu et al. 2021 Xu et al. This content is distributed under the terms of the Creative Commons Attribution 4.0 International license.

10.1128/mBio.03132-20.5 FIG S4 Neutralizing effect of HBoV1 IgG in permissive human airway epithelial (HAE) cell culture (Cufi line). Viral DNA or mRNA copies per million human housekeeping DNA or mRNA copies are shown as single replicates. Note the logarithmic scales on the *y* axes. Download FIG S4, TIF file, 1.8 MB. Copyright © 2021 Xu et al. 2021 Xu et al. This content is distributed under the terms of the Creative Commons Attribution 4.0 International license.

### Endosomal localization of HBoV1 VLPs in B cells.

**FIG 6:**
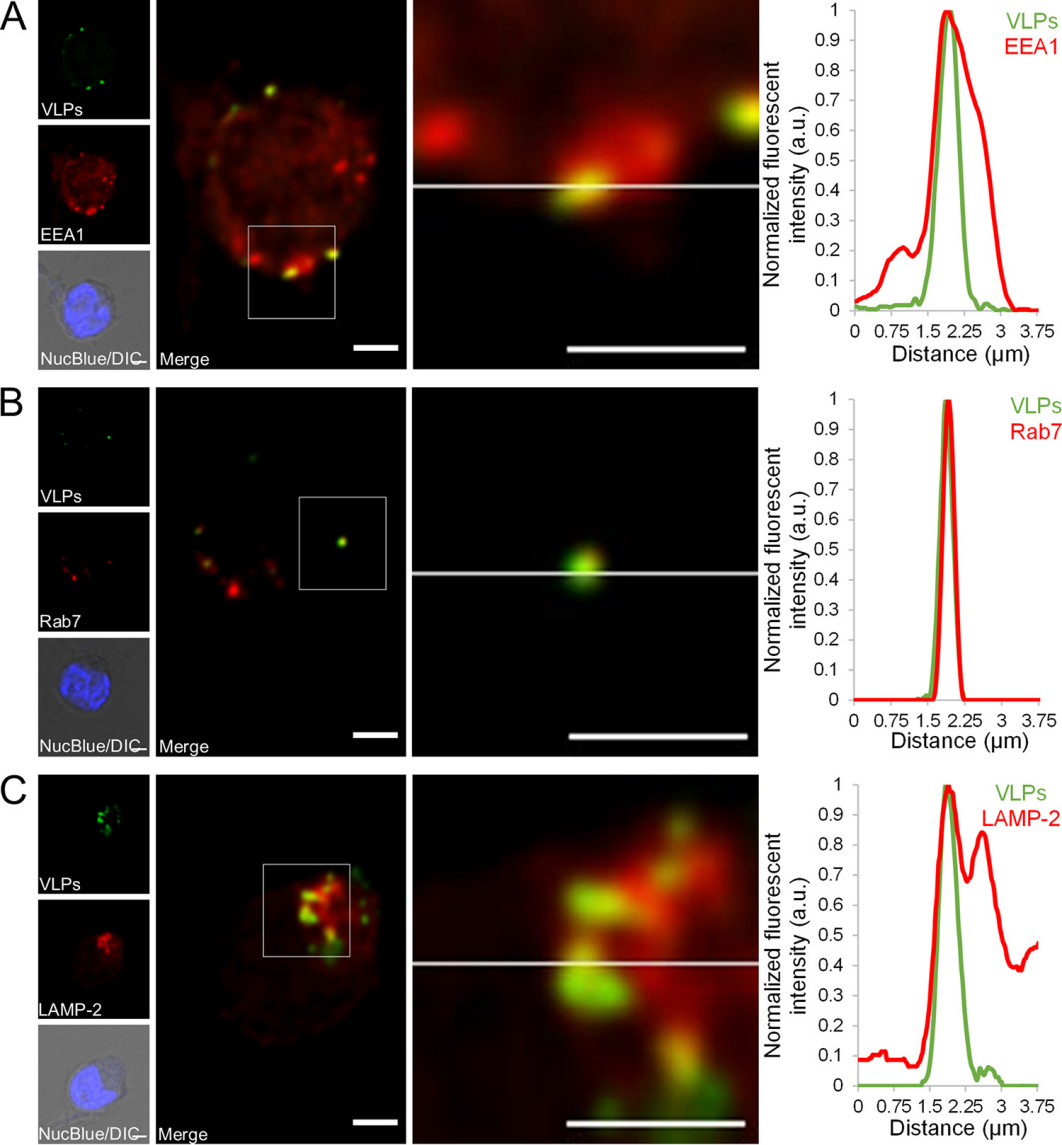
Intracellular distribution of HBoV1 VLPs and endosomal markers. (A to C) Confocal imaging of HBoV1 VLPs-Alexa Fluor 488 (green) localization (arrow) within early endosomes (A), late endosomes (B), and lysosomes (C). Representative optical sections of GM12878 cells incubated for 20 h with HBoV1 VLPs in the presence of a positive serum pool. Close-up images show the regions where HBoV1 VLPs are localized within early endosomes, late endosomes, or late endosomes/lysosomes, positive for EEA1, Rab7, or LAMP-2 markers, respectively. Differential interference contrast (DIC) images merged with NucBlue staining (blue) are also shown. Normalized fluorescence intensity line profiles of the intensity of VLPs (green) together with endosomal and lysosomal markers (red) from the close-up regions are shown beside each image. Normalized fluorescence intensity is shown in arbitrary units (a.u.). Bars, 2 μm.

To study how viral capsids enter the cell, GM12878 B cells were exposed to fluorescent virus-like particles (VLPs) together with HBoV1-specific IgG. Confocal fluorescence imaging showed specific intracellular localization of Alexa Fluor 488-labeled VLPs (Alexa Fluor 488-VLPs) in both early and late endosomal (EEA1, Rab7) (Fig. 6A and B) as well as late endosomal/lysosomal (LAMP-2) (Fig. 6C) marker-associated cytoplasmic vesicles.

### Viral activity in ADE-infected B cells and monocytes.

**FIG 7:**
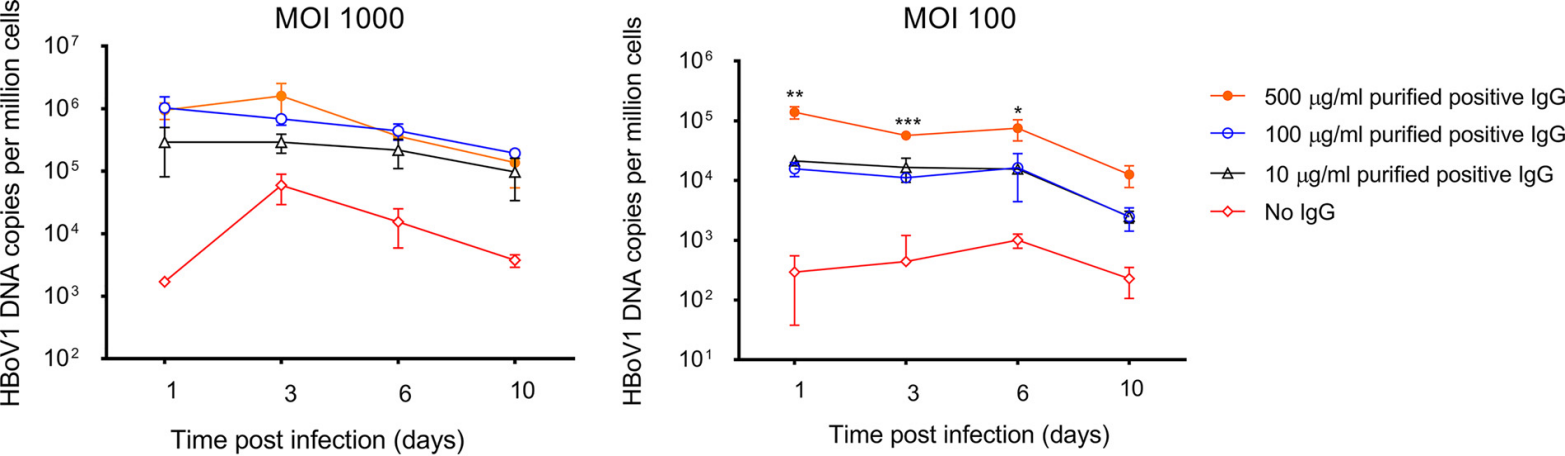
Follow up of HBoV1 ADE infected Raji B cells. Cells were infected at a multiplicity of infection (MOI) of 100 and 1,000 viral genomes per cell in the presence of various concentrations of purified total IgG with HBoV1 reactivity. Viral DNA copies were quantified at days 1, 3, 6, and 10 postinfection. Triplicates are shown. Statistical significance by Student’s *t* test is indicated as follows: *, *P* < 0.05; **, *P* < 0.01; ***, *P* < 0.001. Note that the *y* scale is logarithmic.

In follow-up cultures of HBoV1 ADE-infected Raji B cells for up to 10 days, no net increase in DNA load was seen by qPCR intracellularly (Fig. 7) or in the cell culture medium (data not shown), suggesting that the infection was nonproductive.

**FIG 8:**
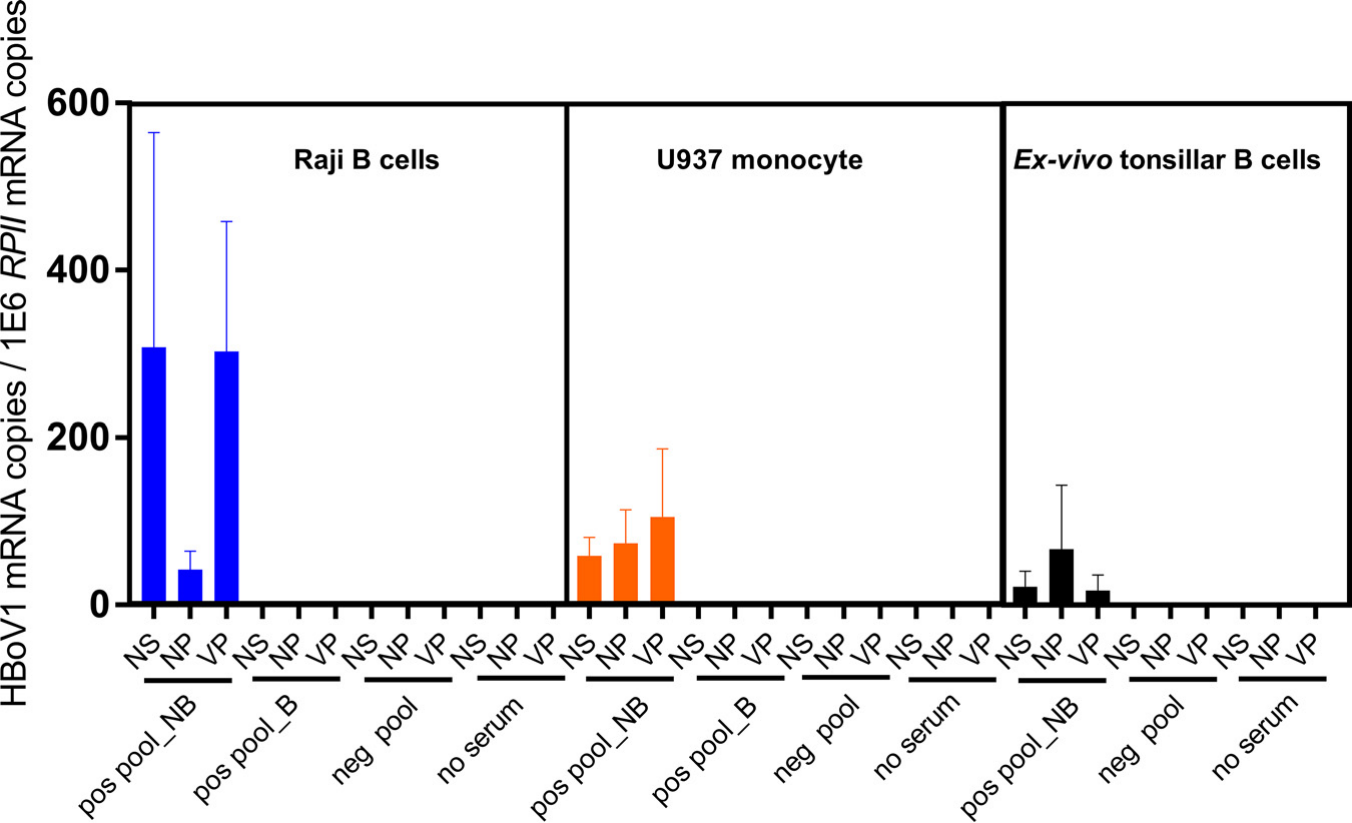
Viral mRNA expression in Raji B cells, U937 monocytes, and *ex vivo*-cultured tonsillar B cells. The infection took place for 24 h in the presence of HBoV1 IgG-positive or -negative serum pools (heat-inactivated serum; 1:100 diluted) or without serum. For FcγRII blocking, cells were preincubated with anti-FcγRII (CD32) antibodies before infection, four replicates are shown. Viral *NS1*, *NP1*, and *VP* mRNAs were quantified by reverse transcription-quantitative PCR (RT-qPCR), and viral mRNA copies per million human reference mRNA, *RPII*, copies are presented. NB, FcγRII not blocked; B, FcγRII blocked; pos pool, HBoV1 IgG-positive serum pool; neg pool, HBoV1 IgG-negative serum pool.

Viral mRNAs were analyzed in ADE-infected *ex vivo*-cultured tonsillar B cells, as well as U937 monocyte and Raji B-cell cultures. As a result, all three (*NS1*, *VP*, and spliced *NP1*) mRNA transcripts were detected in the presence of HBoV1 IgG-positive but not of HBoV1 IgG-negative sera (Fig. 8). In ADE-infected monocytes and Raji B cells, viral mRNAs were undetectable after blocking of FcγRII (Fig. 8). The DNase I-treated RNA preparations without reverse transcriptase (no-RT controls) were PCR negative, and the PCR products amplified from the spliced *NP1* transcripts were confirmed by sequencing to have the correct splicing site.

## DISCUSSION

HBoV1 causes pediatric acute RTI, otitis media, and encephalitis and is able to persist for months both in the airways, hampering PCR diagnosis, and in tonsil tissue (2–7, 11–17, 27–31). Tonsils could thus be reservoirs for virus excretion and spread. How and where HBoV1 is able to infect and persist in the tonsils are not known. We investigated, among nonviremic children with long-term HBoV1 immunity, where in tonsillar tissue HBoV1 resides and whether the persisting virus is active or dormant. We detected viral DNA in adenoid tonsils exclusively in the GCs, mostly in B cells and monocytes, and in a proportion of T cells. Among the B cells, HBoV1 DNA persisted particularly in naive, activated, and memory B-cell subpopulations, albeit in one high-viral-load adenoid, also in plasmablasts. We further showed, in cultured tonsillar primary B cells, B-cell lines and monocytes, but not in T cells, that HBoV1-specific IgG strongly enhanced virus uptake leading to nuclear mRNA transcription and that this effect was inhibited by blocking the cellular FcγRII.

Among other parvoviruses, the highly pathogenic Aleutian mink disease parvovirus and human parvovirus B19 have, in the presence of antibodies, been shown to internalize via FcγR or the complement C1q receptor (CD93) into macrophages, endothelial cells, or B cells, resulting in the establishment of persistent infection (23–26). Similarly, the FcγR-mediated pathway of HBoV1 in our B-cell and monocyte cultures did not lead to complete digestion of the virus for antigen presentation, instead the virus persisted and at least some of them reached the nucleus, as evidenced by *NS*, *NP*, and *VP* mRNA transcription. This endocytic pathway resembles the classical receptor-mediated endocytosis, shown for another bocavirus (bovine parvovirus [BPV]), and could thus be exploited by viruses (32–35). Indeed, in cultured B cells, we observed fluorescent HBoV1 VLPs to localize in early and late endosomes, in concordance with both the FcγRII-mediated immune complex internalization route and the infection route of many parvoviruses; e.g., canine parvovirus has been shown to exploit the cellular endocytic pathway to initiate infection (34, 35).

The specific *in vivo* host cells, receptors, and uptake pathways for HBoV1 are unknown. The virus is capable of productively infecting polarized pseudostratified epithelial cells, mimicking the *in vivo* milieu of the airway epithelium and causing cell hypertrophy and loss of cilia (2, 21, 22). HBoV1 DNA is very prevalent (up to 56%) in tonsillar tissues of children with tonsillitis or hypertrophy without symptoms of RTI (11–18). A common treatment for such ailments is removal of adenotonsillar tissue, which has been shown to decrease the prevalence of particularly DNA viruses, like HBoV1 and adenoviruses, in the nasopharynx (19–20). HBoV1 persistence in tonsils, by itself or in synergy with other microbes, could perhaps trigger and/or maintain an inflammatory response in the tonsils leading to chronic tonsillitis or hypertrophy.

Interestingly, HBoV1 DNA has been detected significantly more often in adenoid tonsils than in palatine tonsils (12, 17). We likewise found HBoV1 DNA in only a single adult’s palatine tonsil, as opposed to 54% of adenoid and 21% of palatine tonsils from children. In the two patients with both types of tonsils available, the viral loads in adenoids were 24- and 5,900-fold higher. Furthermore, we were able to visualize by RISH, HBoV1 DNA in 4/7 adenoid as opposed to 1/7 palatine PCR-positive tonsils. Altogether, these data suggest that adenoids could be a more favored persistence site for HBoV1 than palatine tonsils. In contrast, parvovirus B19 has been shown to persist for life in the palatines (26). Perhaps the infrequency of HBoV1 persistence among adults could reflect HBoV1’s apparent preference for adenoids, which atrophy in adulthood. A reason for this predilection might be that adenoids are covered by pseudostratified ciliated epithelium, whereas palatine tonsils have stratified squamous epithelium. In our study, on the other hand, no viral nucleic acids were seen by RISH beyond follicular GCs, ruling against epithelial persistent infection, even if not against transient infection.

Even if HBoV1 has been shown to often persist in tonsils, the mRNA data revealing viral activity, have been inconclusive (17, 18). In our study, HBoV1 DNA persisted in pediatric adenotonsillar tissues at high prevalence; however, the diverse and often low viral loads (10^0^ to 10^6^ copies/10^6^ cells) would rule against major productive replication. This was corroborated by the infrequent mRNA transcription. Analogously, we saw no net increase in viral DNA loads in B cells cultured for up to 10 days post-ADE, pointing to nonproductive infection. Nevertheless, the RNase-resistant HBoV1 RISH signals of our GCs revealed the presence of both the positive and negative DNA strands and of both the left and right sides of the genome. Because human chromosomal DNA is undetected by RISH, the observed viral DNA could not have been integrated. The positive-strand DNA must thus have originated from the 5% input virions or from dsDNA after complementary strand synthesis or both. Its RISH signal was indeed weaker than that of the negative-strand DNA, as expected of a predominantly negative-strand DNA virus. The typical high particle-to-infectivity ratio in parvovirus infections makes it difficult to verify low-level replication in tissues. Nevertheless, having entered the nucleus at any cell cycle phase, HBoV1 is able to utilize the cellular DNA repair polymerase primed by its own genome’s 3′ hairpin terminus in order to synthesize the complementary strand (36). Indeed, by RT-PCR we detected unspliced *NS1* mRNA (the no-RT control being negative, ruling out interfering DNA amplification) in one adenoid tissue and spliced HBoV1 mRNAs in cultures of ADE infected Raji B cells, monocytes, and *ex vivo* tonsillar B cells. Since transcription initiation can occur only from dsDNA and the cellular polymerases are located exclusively in the nucleus, the presence of viral mRNA confirms that the ssDNA must have been converted into transcribable dsDNA and that the virus has reached the nucleus (36). The mRNA-positive adenoid tissue had the highest viral load by qPCR (2.9 × 10^6^ copies/10^6^ cells), permitting the detection of even low-level NS1 transcripts. Parvoviral NS1 is a cytotoxic multifunctional protein with endonuclease, ATPase, and helicase activities, indispensable for virus replication, but which can be expressed also in nonpermissive cells (2). It has furthermore been shown to transactivate several host genes and to induce cell cycle arrest, DNA damage response, and programmed cell death. Although it is not known whether NS1 is a prerequisite for HBoV1 persistence, the protein may be required for viral genome maintenance in dividing cells. The 3-year-old child, with the mRNA-positive adenoid tissue, was HBoV1 IgG positive (but HBoV2 and -3 IgG negative) yet lacked antiviral IgM and viremia, ruling out acute HBoV1 infection. He underwent adenotonsillectomy due to tonsillar hypertrophy and had an underlying genetic disorder, congenital insensitivity to pain with anhidrosis (CIPA), which is not generally associated with abnormal virus infections (37–39).

Contrary to B19V triggering apoptosis, acute HBoV1 infection of permissive HAE cultures was recently shown to inhibit apoptosis but to promote the pyroptotic inflammasome-induced pathway involving caspase 1 (2, 40). Unlike apoptosis, pyroptosis results in plasma membrane rupture, release of intracellular components, and virus spread. The HBoV1 airway persistence could thus be due to tonsillar excretion of either encapsidated or naked viral DNA or to migratory virus-containing lymphocytes or macrophages. It has been suggested that viruses spread from the tonsils to the middle ear, sinuses, or lungs, causing inflammation (27–31). In intestinal tissue, bocavirus DNA has been shown to persist as episomes and to be excreted in feces, pointing to an analogy with tonsils and airways (41, 42).

We showed that HBoV1 is retained in adenotonsillar GC B cells as dormant or weakly transcribing viral genomes and that the virus in cultured tonsillar B cells is taken up via ADE, likewise leading to mRNA transcription. To which extent this is a common feature among tonsil-persistent viruses is not known. At least the HBoV1 B-cell persistence, preferring pediatric adenoids, is dissimilar to that of parvovirus B19V, preferring adult palatine tonsils (26). Moreover, adenovirus has been shown to persist in tonsillar T cells, rather than B cells, with very low viral activity, yet it could be reactivated upon *in vitro* lymphocyte stimulation, resulting in infectious virus production (43, 44). Recently, silent influenza A virus, found in both B and T cells, could be rescued from tonsillar persistency (45). Likewise, the quiescent HBoV1 genomes, even though not as easily culturable, might be able to reactivate and replicate or translate proteins with lytic, anti-apoptotic, pyroptotic, or inflammatory potential. A few studies describe putative reactivations of HBoV1 persistence (7–10). Moreover, IgG-level fluctuations have been observed during follow-up of children and adults, possibly implying reactivation or reinfection of HBoV1 or infection by the related HBoV2 or HBoV3 (46, 47). These enteric HBoVs are known to interfere with HBoV1 serology via cross-reactivity and original antigenic sin (OAS), an immunological phenomenon first described for dengue and influenza viruses, in which a prior infection interferes with the antibody production to a subsequent infection by a related virus (47–50). It is tempting to speculate that HBoV1 persistence in GCs might regulate the FcγRII-mediated B-cell selection process leading to OAS (51). Analogously, Epstein-Barr and measles viruses are known to infect B cells via their respective receptors, CD21 and CD150, and to interfere with B-cell immune functions (52, 53). It is possible that preexisting HBoV2 or HBoV3 immunity could mediate the HBoV1 ADE infection of GCs, in line with the subsequent infections by dengue virus serotypes (54–57). In our study, however, only 2 of the 12 HBoV1 DNA-positive children with available serum had prior HBoV2 or HBoV3 immunity, suggesting that this would neither be required nor frequent.

To conclude, we provide the first evidence of HBoV1 DNA persistence in pediatric adenotonsillar GCs, predominantly in B cells and monocytes. Our data point to ADE-mediated endocytic uptake of HBoV1 via FcγRII, leading to nuclear synthesis of complementary strand DNA and mRNA, yet with little or no productive replication. Whether the persistent virus can alter GC functions, be released by cellular pyroptosis accounting for the long-lived PCR positivity in nasopharyngeal mucosa, or be reactivated by other viruses or immunosuppression, must be determined in forthcoming studies.

## MATERIALS AND METHODS

### Patients and tissue preparation.

Tonsillar samples were collected in spring 2015 and in winter 2019 at the Helsinki University Hospital from a total of 41 patients ≤10 years of age (median, 5.96 years) and from 38 patients 10 to 69 years of age (median, 26.5 years), who underwent tonsillar surgery (tonsillectomy, tonsillotomy, or adenoidectomy) for chronic tonsillitis or tonsillar hypertrophy. In 2019, of 14 children, four provided tissue of only adenoids, one of both left and right tonsils, and nine of both adenoids and tonsils. In 2015, 27 children ≤10 years of age and all the 38 patients aged >10 years, provided right-side tonsils only. The patients lacked acute respiratory symptoms during the 2 weeks before surgery. Informed consent was obtained from all patients or children’s parents. The study protocol was approved by the Ethics Committee of the Hospital District of Helsinki and Uusimaa, and institutional research permission was granted by the Department of Otorhinolaryngology, Helsinki University Hospital, Helsinki, Finland.

Tissues collected during 2019 were processed immediately as three distinct preparations (Preps 1, 2 and 3). Two pieces of tonsil, for respective DNA and RNA purification, were immersed immediately after surgery in RNAlater overnight, and then stored at −20°C (Prep 1), while two other pieces were freshly frozen, then embedded in OCT compound, and stored at −80°C (Prep 2). The remaining tissue was placed in cold phosphate-buffered saline (PBS) and stored at +4°C until mechanical homogenization with a syringe plunger. The homogenate was then treated with collagenase, 0.1 mg/ml Liberase TL (Roche), at 37°C for 30 min, and the cell preparations were filtered through a 70-μm nylon mesh, washed twice with PBS, and stored in liquid nitrogen (Prep 3). The tonsillar tissues collected in 2015 were homogenized as Prep 3 above but were not treated with collagenase (26). Serum samples were obtained from all the pediatric patients, except two enrolled in 2015, and studied for HBoV1 DNA and HBoV1-3-specific antibodies.

Details of DNA and mRNA extraction and PCR amplification from tonsillar tissues and cell cultures, as well as HBoV1 virus production are described in Text S1 in the supplemental material. Primers and probes are listed in Table 1.

**TABLE 1:** Primers and probes used for qPCRs in the study

Gene	Primer and probe^*a*^	GenBank accession no.
*NP1* mRNA	5′-CGGCGAGTGAACATCTCTGGA-3′ (203–223)	NC_007455
	5′-TGCTTGTCTTTCATATTCCCT-3′ (2438–2418)	
	5′-FAM-TGTCCACCCAAGAAACGTCGTCTAA-BHQ1-3′	
*NS1* mRNA^*b*^	5′-GTTGGTCACGCCCTGTGCT-3′ (1549–1567)	JQ923422
	5′-AATCCCTTGGACGATTGCCT-3′ (1649–1668)	
	5′-FAM-TTTGCTTTTACGGGCCTGCCTCA-BHQ1-3′	
*VP* mRNA^*b*^ and DNA	5′-TAGACGCACCACAGAACACC-3′ (3579–3598)	JQ923422
	5′-AAGTGAGAACCTCCGACCCA-3′ (3665–3684)	
	5′-FAM-AGGAAGTATTGGAGGAGGAAAAGGAT-BHQ1-3′	
*RPII* mRNA	5′-GCACCACGTCCAATGACAT-3′ (838–856)	X74870
	5′-GTGCGGCTGCTTCCATAA-3′ (1452–1469)	
	5′-FAM-TACCACGTCATCTCCTTTGATGGCTCCTA-BHQ1-3′	
*NS1* DNA	5′-CCTATATAAGCTGCTGCACTTCCTG-3′ (152–177)	NC_007455
	5′-AAGCCATAGTAGACTCACCACAAG-3′ (235–259)	
	5′-FAM-CCAGAG ATGTTCACTCGCCG-BHQ1-3′	
*RNase P*	5′-GAGGGAAGCTCATCAGTGGGG-3′ (9–29)	NR_002312.1
DNA	5′-CCCTAGTCTCAGACCTTCCCAAG-3′ (70–92)	
	5′-FAM-AGTGCGTCCTGTCACTCCACTC-TAMRA-3′	

a FAM, 6-carboxyfluorescein; BHQ, black hole quencher; TAMRA, 6-carboxytetramethylrhodamin.

b Note that as *NS1* and *VP* RT-PCRs are not specific for the major protein-coding mRNAs, alternatively spliced transcripts for the other proteins may also be detected by these primers and probes. For a transcription map of the HBoV1 genome, please see reference 2. NP1, nuclear phosphoprotein 1; NS1, nonstructural protein 1; VP, virus capsid protein; RPII, cellular RNA polymerase II; RNase P, cellular RNase P.

10.1128/mBio.03132-20.1 TEXT S1 Supplemental materials and methods for DNA extraction, qPCRs, RNA extraction, RT-PCRs, B- and T-cell and monocyte proportions in tonsils, cellular Fc receptor staining, HBoV1 virus production, fluorescently labeled virus-like particles (VLPs) and Raji cell uptake, imaging by confocal microscope, and effects of HBoV1 IgG on infection of permissive human airway epithelial (HAE) cells. Download Text S1, PDF file, 0.1 MB. Copyright © 2021 Xu et al. 2021 Xu et al. This content is distributed under the terms of the Creative Commons Attribution 4.0 International license.

### Magnetic positive selection of cell fractions.

To investigate the cell types of HBoV1 persistence, three populations were isolated sequentially from HBoV1 DNA-positive tonsils (Prep 3) using CD antibody-coupled magnetic beads (Dynabeads; Invitrogen), according to the manufacturer’s instructions: T cells with anti-CD3 beads, B cells with anti-CD19 beads for the CD3-depleted cells, and monocytes with anti-CD14 beads for the CD3-CD19-depleted cells. To test the purity of the three isolates, cells from the beads were released with DETACHaBEADS (Invitrogen), and labeled with anti-human CD19-FITC (CD19 labeled with fluorescein isothiocyanate) (clone SJ25-C1; Invitrogen), CD3-PE/Cy5.5 (CD3 labeled with phycoerythrin or Cy5.5) (clone UCHT1; Abcam), or CD14-PE (clone MφP9; BD Biosciences) and analyzed on a BD Accuri C6 flow cytometer as described previously (26). Assessment of the cell fraction proportions is described in Text S1.

### B-cell sorting.

To characterize the B-cell subpopulation(s) harboring HBoV1 DNA, tonsillar cell Prep 3 was sorted with multicolor fluorescence-activated cell sorting (FACS) on a BD Influx Cell Sorter based on cell viability (7-aminoactinomycin D [7AAD]; Invitrogen) and according to the cellular markers (anti-human CD19-PE [clone CB19; Abcam], CD20-PE-Cy7 [clone 2H7; Biolegend], IgD-allophycocyanin [APC]-Cy7 [clone IA6-2; Biolegend], CD27-FITC [clone LT27; Abcam], and CD38-BV421 [clone HB-7; Biolegend]). Four subsets of B cells (CD19^+^) were sorted: naive (CD20^+^ IgD^+^ CD27^−^), memory (CD20^+^ IgD^−^ CD27^+^), and activated B cells (CD20^+^ IgD^+^ CD27^+^) and plasmablasts (CD20^−^ IgD^−^ CD27^+^ CD38^+^), and one aliquot of each cell subset was stored at −20°C for viral DNA detection. The remaining cells were fixed with 4% paraformaldehyde at 37°C, resuspended in 70% ethanol, and cytocentrifuged (800 rpm, 20 min) onto glass slides (SuperFrost Plus), followed by HBoV1 RNAscope *in situ* hybridization (RISH) staining as described below.

### Human serum, purified IgG, and IgG EIA.

Human sera were pooled from 6 individuals with, and from 11 individuals without, HBoV1 IgG reactivity, in two pools. The individual and pooled sera as well as all pediatric sera were tested for HBoV1, -2, and -3 IgG by competitive, and IgM by non-competitive, enzyme immunoassays (EIAs) as previously described (58, 59). The serum pools were examined with or without heat inactivation for 30 min at 56°C. Total IgG was purified from the pools by HiTrap protein A HP (GE Healthcare) columns. Protein concentrations were measured with Pierce BCA (Thermo Fisher Scientific).

### Antibody-dependent enhancement.

To determine antibody-dependent enhancement (ADE), GM04671 (Raji) and GM12878 lymphoblastoid B-cell lines (both from Coriell Institute) and U937 monocyte line (ATCC CRL-1593.2) and *ex vivo* tonsillar B cells were cultured in RPMI, supplemented with 10% fetal bovine serum, and infected with HBoV1 (see Text S1 for virus production) in the presence of purified total IgG or serum, with or without HBoV1 reactivity. Briefly, 2 × 10^5^ cells were cultured on a 96-well plate and infected with HBoV1 particles at a multiplicity of infection (MOI) of 100 viral genome copies per cell for 48 h in the presence of 10 μg/ml of purified total HBoV1-positive or -negative IgG. Infected and uninfected cells without IgG or serum served as controls. After culture, cells were treated with 0.25% trypsin-EDTA (Gibco) for 15 min at 37°C to remove surface-bound virus, washed three times with cold PBS, and suspended in 200 μl PBS for viral DNA and mRNA quantification. ADE with Alexa Fluor-labeled VLPs and neutralization of HBoV1 in HAE cells are described in Text S1.

### Blocking of cellular Fc receptors.

To confirm that HBoV1 IgG enhanced viral uptake through the Fc receptors in two B-cell lines and the monocyte line, a total of 2 × 10^5^ cells were incubated with purified mouse anti-CD32 (clone 3D3; BD Biosciences) for FcγRII, anti-CD64 (clone 10.1; BD Biosciences) for FcγRI, or both, at 10 μg/ml for 1h at 37°C, followed by the ADE assay and qPCR, as described above. The cells were cultured for 40 h, trypsinized, and washed as described above. The presence of Fc receptors on primary B cells and cell lines was assessed by flow cytometry (Text S1).

### Confocal imaging.

For microscopy, GM12878 B cells were incubated with fluorescent HBoV1 VLP-Alexa Fluor 488 for 20 h in the presence of the HBoV1 IgG-positive serum pool (1:100 diluted) and then treated with 500 mM NH_4_Cl for 4 min at room temperature to remove noninternalized surface-bound VLPs. Cells were air dried overnight on high-performance cover glasses (0.17-mm thickness, 18 × 18 mm^2^; Carl Zeiss), fixed with 4% paraformaldehyde for 15 min at room temperature, and permeabilized with 0.1% Triton X-100 in PBS supplemented with 0.5% bovine serum albumin (BSA) and 0.01% sodium azide for 15 min. Early endosomes, late endosomes, and late endosomes/lysosomes were visualized with primary mouse or rabbit antibodies against EEA1 (ab2900; Abcam), Rab7 (ab137029;Abcam) and LAMP-2 (9840-01 mouse anti-human CD07b-UNLB; DSHB Developmental Studies Hybridoma Bank), respectively. The primary antibodies were followed by secondary Alexa Fluor 555-conjugated goat anti-mouse and goat anti-rabbit antibodies (Thermo Fisher Scientific) and mounted in ProLong Glass Antifade Mountant with NucBlue (Invitrogen) and imaged as described in Text S1.

### RNAscope *in situ* hybridization and immunohistochemistry.

To identify the adenotonsillar persistence site, RNAscope ISH (RISH) technology (Advanced Cell Diagnostics [ACD], Newark, CA) was used for viral RNA or DNA detection. Fresh-frozen tonsils in OCT were cut into 20-μm-thick sections in a cryostat at −20°C, dried on glass slides (SuperFrost Plus) at −20°C for 1 h, and fixed with 4% paraformaldehyde for 1 h on ice. HBoV1 nucleic acid was detected using paired double-Z oligonucleotide probes against target HBoV1-VP3 positive-sense DNA or mRNA (RNAscope Probe-V-HBoV1-VP, 28 to 1,001 bp; 20 pairs; GenBank accession no. KF385975.1) and HBoV1-NS negative-sense DNA (RNAscope Probe-V-HBoV1-NS-sense, 1,152 to 2,267 bp, 20 pairs; GenBank accession no. JQ923422.1). RNAscope 2.5 HD reagent kit-RED (PN 322350; ACD) was employed according to the manufacturer’s protocol, presenting the targets as red dots. Hybridization was done in an ACD HybEZ II oven. Probes targeting the human housekeeping gene *PPIB* and the bacterial gene *dapB* (ACD) served as positive and negative controls, respectively. To differentiate between viral DNA and mRNA signals, RNase A (5 mg/ml, 40°C for 30 min; Qiagen) was applied to the sections before RISH.

For cell typing, a B-cell marker (mouse anti-human CD20 monoclonal antibody [mAb], clone L26, Biocare Medical) was employed after RISH, by chromogenic immunohistochemistry (IHC) using the MACH1 Universal HRP-Polymer detection kit (Biocare Medical). Sections were counterstained with hematoxylin and mounted with VectaMount medium (Vectorlabs).

Bright-field images were generated with a 40× objective with extended focus using 3DHISTECH Pannoramic 250 FLASH II digital slide scanner at the Genome Biology Unit, supported by HiLIFE and the Faculty of Medicine, University of Helsinki, Finland.

### Statistical analysis.

Student’s *t* test was used for analysis of statistical significance of viral loads in two independent groups. These analyses were conducted using GraphPad Prism 7 software (San Diego, CA). Differences were considered statistically significant at *P* < 0.05.
