# Supplementary figures and images for: Persistence of Human Bocavirus 1 in Tonsillar Germinal Centers and Antibody-Dependent Enhancement of Infection
Source: mBio. 2021 Feb 2;12(1):e03132-20. doi: 10.1128/mBio.03132-20 (PMC7858059; doi:10.1128/mBio.03132-20)

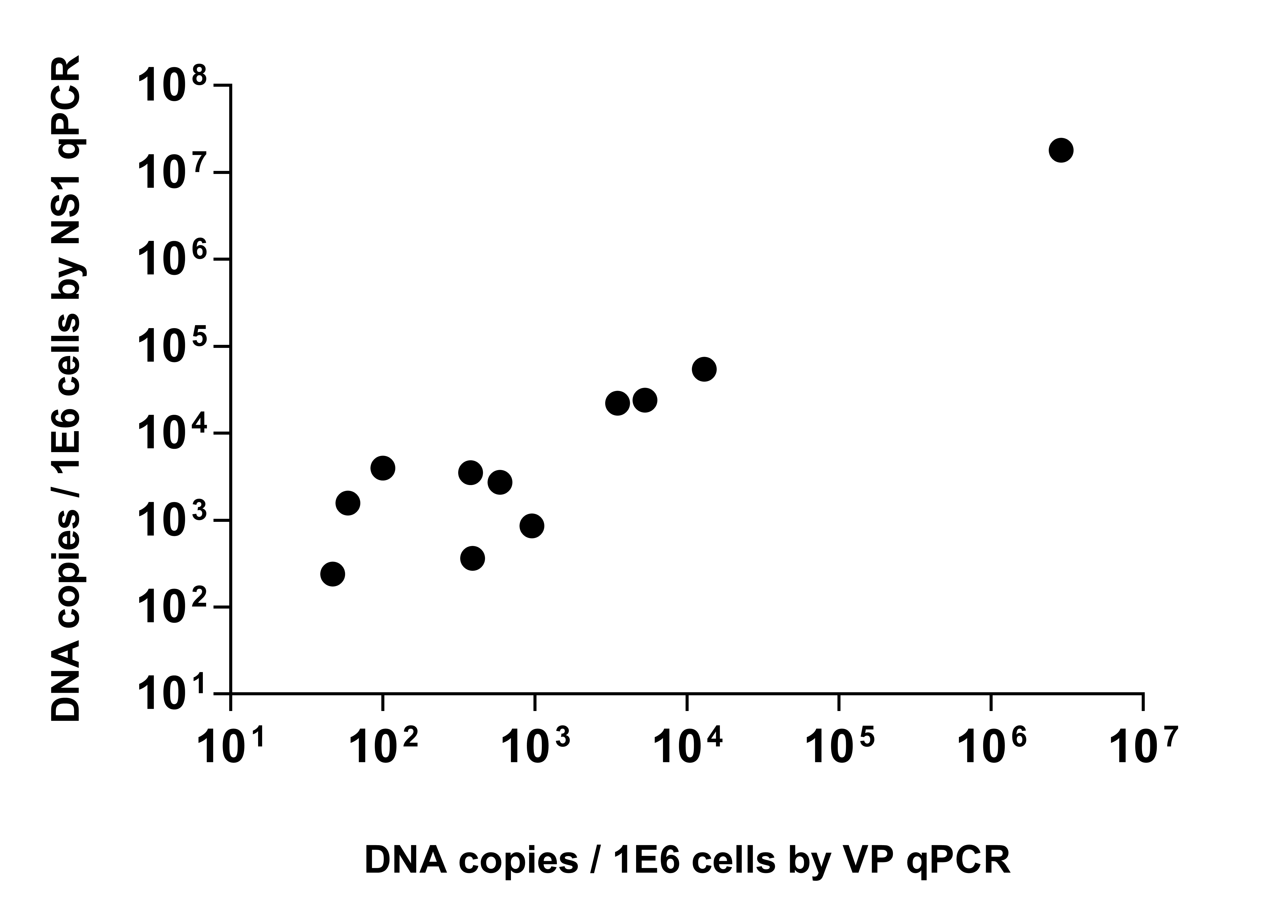

Supplement: FIG S1 [file mBio.03132-20-sf001.tif]

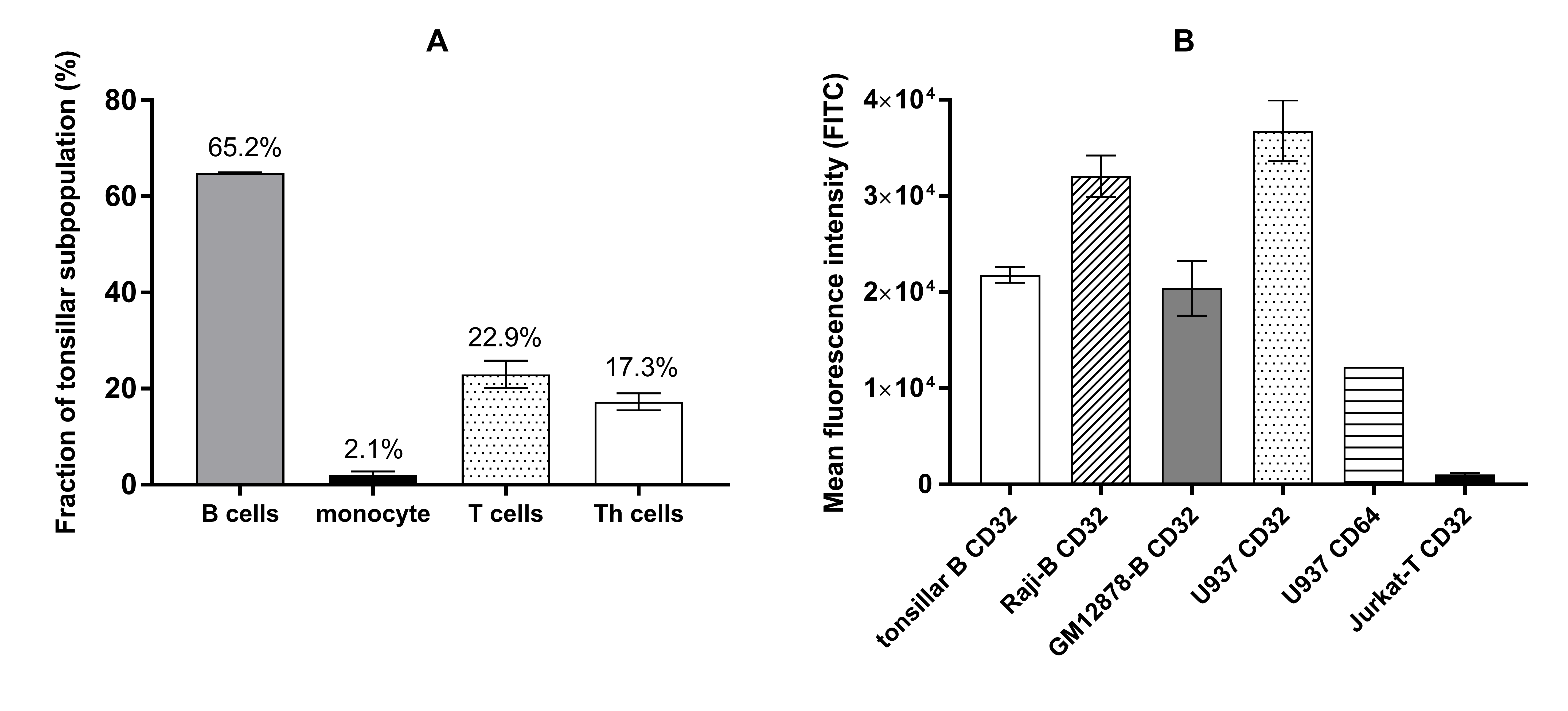

Supplement: FIG S2 [file mBio.03132-20-sf002.tif]

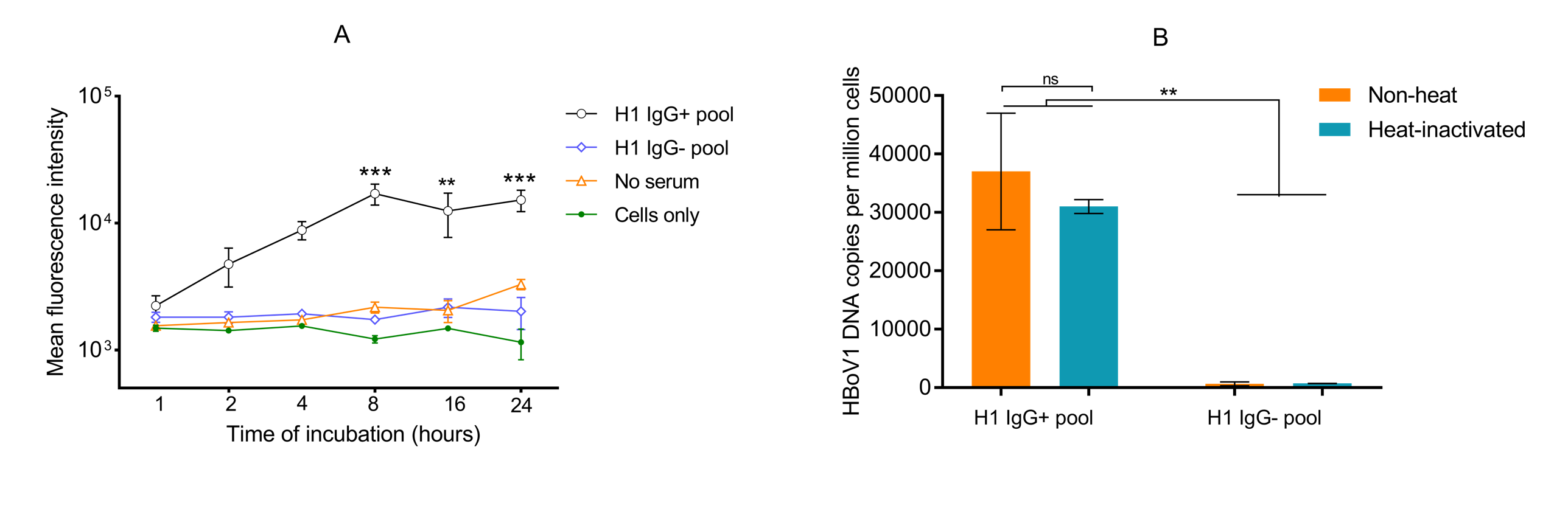

Supplement: FIG S3 [file mBio.03132-20-sf003.tif]

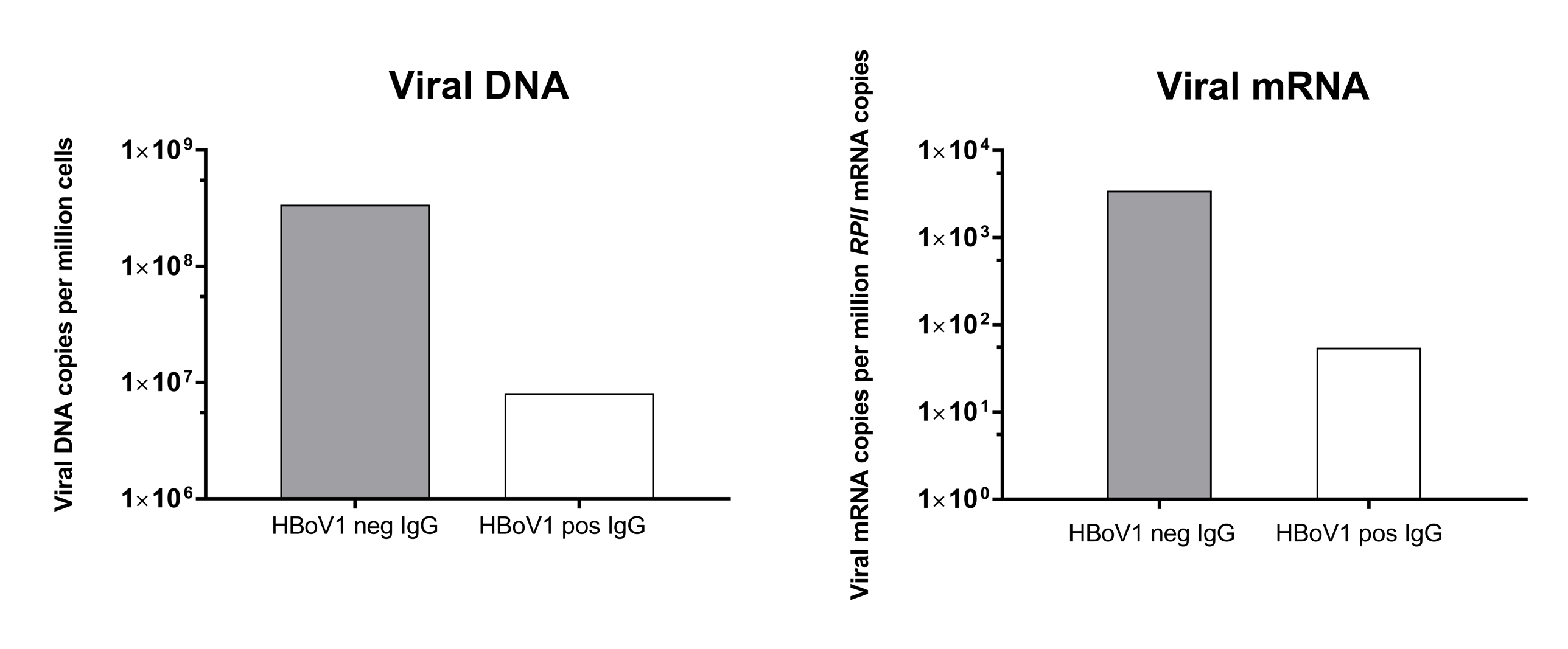

Supplement: FIG S4 [file mBio.03132-20-sf004.tif]
