# Supplementary material for: Persistence of Human Bocavirus 1 in Tonsillar Germinal Centers and Antibody-Dependent Enhancement of Infection
Source: mBio. 2021 Feb 2;12(1):e03132-20. doi: 10.1128/mBio.03132-20 (PMC7858059; doi:10.1128/mBio.03132-20)
Supplement: TEXT S1 [file mBio.03132-20-s0001.pdf]

DNA extraction, qPCRs. Total DNA was extracted from total tonsillar tissues (Prep 1), magnetically separated tonsillar cell suspensions from Prep 3, FACS-sorted B cells, and cell cultures, with the QIAamp DNA Mini Kit (Qiagen), or for the 2015 tonsils, with the KingFisher Cell and Tissue DNA kit in an automated KingFisher Duo Purification system (1), following the manufacturers' manuals. HBoV1 DNA was quantified by in-house quantitative PCR (qPCR), targeting the *NS1* gene of the HBoV1 genome, as described (2). Ten-fold-diluted plasmids, containing the near-full length HBoV1 genome (2) or the human *RNase P* gene (a kind gift from Dr. Janet Butel (3)), served as quantification standards. The human single-copy gene *RNase P* was quantified to normalize the viral DNA copies to one million human cells (4). All samples were analyzed in duplicates by qPCR. To further verify that not only the left side (NS1) of the genome is present, we screened extracted DNAs of 11 tonsils with another qPCR, amplifying the right side (VP), with primers and probe for VP mRNA PCR. Comparison of the two qPCRs is shown in Fig. S1.

RNA extraction, RT-PCRs. Total RNA from tonsil Prep 1 was isolated with RNeasy Mini Kit (Qiagen) and treated with DNase I for 15 min to remove contaminating genomic DNA. Total RNA and DNA from cell cultures were isolated with QIAamp cador Pathogen Mini Kit (Qiagen) for both viral DNA and mRNA detection. The RNA/DNA preps for mRNA detection were treated with DNase I for up to 1h to remove genomic DNA. HBoV1 mRNAs were detected as described (5), by a two-step reverse transcription (RT) -qPCR comprising M-MLV Reverse Transcriptase (Life Technologies) and random hexamers (Promega), followed by qPCR targeting the *NS1* or *VP* gene or the spliced *NP1* mRNA. The amplification curves were analyzed by AriaMx 1.6 software (Agilent Technologies), and the Cts were determined as 10 times the standard deviation of the fluorescence baseline from cycles 5 to 9. RNA or DNase I-treated RNA yet untreated with reverse transcriptase served as no-RT control in the qPCR. Human housekeeping mRNA, *RNA polymerase II (RPII)*, served as positive control. All PCR primers and probes used in this study are listed in Table 1.

B- and T-cell and monocyte proportions in tonsils. Tonsillar B- and T-cell, and monocyte fractions were assessed with distinct cellular markers and analyzed with FACS on BD Influx Cell Sorter. CD19-PE (LT19, Immuno Tools), CD3-BV711 (Clone SK7, BD Bioscience), CD4-FITC (Clone RPA-T4, BD Bioscience), CD14-APC (Clone 61D3, Invitrogen), were used to identify B cells, T cells, Th cells, and monocytes, respectively. Results shown in Fig. S2A.

Cellular Fc receptor staining. The abundancy of cellular Fc receptors on primary B cells, cultured B- or T-cells, or monocytes was assessed by flow cytometry. Briefly, 1×10^6 cells were incubated with 1:100 diluted mouse anti-CD32 (Clone 3D3, BD Biosciences) for FcγRII, or with anti-CD64 (Clone 10.1, BD Biosciences) for FcγRI on ice for 30 min, followed by 1:100 diluted goat anti-mouse IgM/IgG secondary antibody. Fluorescence intensity was analyzed on a BD Accuri C6 Flow Cytometer. Results are shown in Fig. S2B.

HBoV1 virus production. HBoV1 virions were produced in HEK293 cells transfected with plasmid pIHBoV1 containing the full-length viral genome (6). Briefly, HEK293 cells were seeded on a 15-mm Petri dish and transfected with 15µg pIHBoV1 using the Lipofectamine 2000 transfection reagent (Invitrogen). Cell pellets were collected after 48 hours in PBS and lysed with 4 cycles of freezing (in liquid nitrogen) and thawing (37°C water bath). Virus was purified from the supernatant by CsCl gradient ultracentrifugation for 16 hours at 4°C, at 32 000 rpm (Beckman, CA, USA), and continuous fractions of 1 ml were collected and dialyzed against PBS. Viral copies of each fraction were quantified by qPCR as described above, and the fractions with the highest viral loads were used.

Fluorescent virus-like particles (VLPs) and Raji cell uptake. HBoV1-VP3 VLPs, produced with the Baculovirus Expression System (7), were fluorochrome labelled with the Alexa Fluor 488 Protein Labeling Kit (Invitrogen) as per manufacturer's protocol. Virus uptake in B cells was monitored using VLP-Alexa 488. Raji cells were incubated with 60ng VLP-Alexa 488 in the presence of HBoV1 IgG-positive or -negative serum pools (heat inactivated; 1:100 diluted). Cells were trypsinized and washed. Mean fluorescence intensity was measured after 1h, 2h, 4h, 8h, 16h and 24h of culture on a BD Accuri C6 Flow Cytometer. Results are shown in Fig. S3.

Imaging by confocal microscopy. The immunolabelled cells were imaged using Nikon A1R laser scanning confocal microscope (Nikon Instruments Inc.) with CFI Plan Apo VC 60XH oil immersion objective (N.A 1.4). NucBlue was excited with a 405 nm diode laser, Alexa 488 and Alexa 555 with 488 nm and 561 nm Argon lasers, respectively. NucBlue fluorescence was detected with a 450/50 nm band-pass filter and Alexa 488 fluorescence with a 515/30 nm band-pass filter. A 561 nm sapphire laser was used to excite Alexa 555, and the fluorescence was collected with a 595/50 nm band-pass filter. Stacks of 256x256 pixels were collected with a size of 60 nm/pixel in the x- and y- directions, and 150 nm in the z-direction. Images were iteratively

deconvoluted with Huygens Essential software (SVI) with a signal-to-noise ratio of 5 and a quality threshold of 0.05.

Effects of HBoV1 IgG on infection of permissive human airway epithelial (HAE) cells. Cufi cells were infected at MOI of 10^4 copies/cell in the presence of 200 μg/ml purified IgG from HBoV1 IgG-positive or -negative serum pools. Cells were collected on day 6 post infection and viral DNA and NP mRNA were measured with qPCR and RT-qPCR, respectively. Results are shown in Fig. S4.

References

1. Pyöriä L, Toppinen M, Mäntylä E et al. 2017. Extinct type of human parvovirus B19 persists in tonsillar B cells. Nat Commun 8:14930.
2. Kantola K, Sadeghi M, Antikainen J et al. 2010. Real-time quantitative PCR detection of four human bocaviruses. J Clin Microbiol 48:4044–4050.
3. McNees AL, White ZS, Zanwar P et al. 2005. Specific and quantitative detection of human polyomaviruses BKV, JCV, and SV40 by real time PCR. J Clin Virol 34:52–62.
4. Sadeghi M, Aaltonen LM, Hedman L et al. 2014. Detection of TS polyomavirus DNA in tonsillar tissues of children and adults: evidence for site of viral latency. J Clin Virol 59:55–58.
5. Xu M, Arku B, Jartti T et al. 2017. Comparative diagnosis of human bocavirus 1 respiratory infection with messenger RNA reverse-transcription polymerase chain reaction (PCR), DNA quantitative PCR, and serology. J Infect Dis 215:1551–1557.
6. Huang Q, Deng X, Yan Z et al. 2012. Establishment of a reverse genetics system for studying human bocavirus in human airway epithelia. PLoS Pathog 8:e1002899.
7. Söderlund-Venermo M, Lahtinen A, Jartti T et al. 2009. Clinical assessment and improved diagnosis of bocavirus-induced wheezing in children, Finland. Emerg Infect Dis 15:1423–1430.
